# The Status of Cardiovascular Health in Rural and Urban Areas of Janów Lubelski District in Eastern Poland: A Population-Based Study

**DOI:** 10.3390/ijerph15112388

**Published:** 2018-10-28

**Authors:** Grzegorz Józef Nowicki, Barbara Ślusarska, Honorata Piasecka, Agnieszka Bartoszek, Katarzyna Kocka, Alina Deluga

**Affiliations:** Department of Family Medicine and Community Nursing, Medical University of Lublin, Staszica 6 Str., PL-20-081 Lublin, Poland; basiaslusarska@gmail.com (B.Ś.); honorata_p@onet.eu (H.P.); agabartoszek@wp.pl (A.B.); katarzyna48@op.pl (K.K.); alina_deluga@poczta.onet.pl (A.D.)

**Keywords:** ideal cardiovascular health, cardiovascular disease, health status, prevention and control, Poland

## Abstract

Ideal cardiovascular health (CVH) has been defined by the American Heart Association as the lack of cardiovascular disease and the presence of seven key factors and health behaviors. In this study, we aimed to estimate the prevalence of ideal and poor CVH among the Polish adult population based on the example of the inhabitants of Janów district in Lubelskie Voivodship, taking the chosen socio-demographic factors into consideration. This is a cross-sectional study conducted among 3901 adults without cardiovascular diseases, aged between 35 and 64 years. Participants completed a questionnaire, and they had anthropometric and physiological measurements taken. Blood samples were analyzed for fasting glucose and cholesterol levels. Ideal CVH was found in 5.4% of the participants, with the advantage of being toward city dwellers over those living in the rural areas (6.3% vs. 5.0%) *p* = 0.02. In the case of the residents of rural areas, their likelihood of having an ideal body mass index (BMI) was found to be 22% lower (odds ratio (OR) = 0.78; 95% CI: 0.66–0.92), their likelihood of having an ideal diet was found to be 27% lower (OR = 0.71; 95% CI: 0.54–0.94), their likelihood of having perfect blood pressure was found to be 29% lower (OR = 0.71; 95% CI: 0.56–0.89), and their likelihood of having the perfect glucose levels was found to be 28% lower (OR = 0.72; 95% CI: 0.63–0.84), than the residents of urban areas. The prevalence of ideal cardiovascular behaviors and factors is lower in the rural community compared with people living in the city. Results indicate that more effort should be dedicated toward the country’s health policy, specifically concerning primary prevention. Preventive actions in the field of cardiovascular disease should be addressed to the residents of rural areas to a larger extent.

## 1. Introduction

Cardiovascular disease (CVD) is one of the primary causes of death around the world, despite the widespread implementation of preventive strategies and the development of effective therapies [1,2]. It was estimated that in 2013, approximately 17.3 million people died of CVD, with 80% of these deaths occurring in countries with a low or average income. On the contrary, in developed countries with high income, there was a noticeable decrease in the death rate caused by ischemic heart disease and cerebrovascular accidents [3,4]. It is estimated that 4 million people die in Europe yearly due to CVD [5]. Poland is classified as high-risk mortality country due to CVD (i.e., mortality of CVD >450/100,000 in men and >350/100,000 in women) [6]. In addition, in Poland mortality due to CVD in rural areas is about 20% higher than in urban areas [7,8].

Preventive measures implemented at the level of the general population, promoting a healthy lifestyle, as well as at the level of individual engagements, i.e., with those burdened with moderate to high CVD risk or patients with diagnosed CVD, play a significant role in preventing the development of cardiovascular diseases by modifying unhealthy style of life [6]. The results of the Coronary Artery Risk Development In Adult (CARDIA) study suggest that 80% of CVS incidents can be avoided by optimizing the control of risk factors and adjusting the lifestyle, including changing the diet [9]. The European Society of Cardiology (ESC), in the guidelines for the prevention of cardiovascular disease, emphasizes the need for lifestyle changes in the prevention of cardiovascular risk. The ESC sets goals aimed at modifying risk factors in clinical practice to which the patient should strive to minimize cardiovascular risk; these risk factors include: smoking (no exposure to smoke in any form), diet (low in saturated fat, containing whole grains, vegetables, fruit and fish), physical activity (≥150 min/week of moderate effort 30 min for 5 days per week or 75 min/week of vigorous physical activity or combination of the above), normal lipogram and blood glucose values, as well as body weight and perimeter belt (BMI 20–25 kg/m^2^, waist circumference <94 cm, men or <80 cm in women). Moreover, the ESC recommends the use of cards Systemic Coronary Risk Estimation (SCORE) for the assessment of the overall cardiovascular risk in clinical practice [6]. The ESC strategy for the prevention of cardiovascular disease focuses on reducing the risk factors.

In 2010, the American Heart Association (AHA) set itself the goal of improving cardiovascular health (CVH) by 20% in the American population by the year 2020. The concept of ideal cardiovascular health (ICH) was then created, the indicators of which allows to control and set strategic actions with respect to the practice of preventive cardiology. The defined criteria of ICH are based on the following 7-elements tool: four behavioral factors (smoking, physical activity, BMI, and diet) and three biological factors (blood pressure, blood glucose, and cholesterol levels). The level of maintaining particular components sets a CVH plane covering three categories: poor, intermediate, and ideal [4]. This approach emphasizes primary prevention, in which effort is directed toward preventing the development of behavioral risk factors, which is the opposite of the secondary prevention, which focuses mainly on the occurrence or reoccurrence of CVD [10]. Research has confirmed that the number of ideal factors of CVH is a strong indicator, one which is inversely proportional to mortality in American [11] and Chinese populations [12] and the changes in arterial vessels linked to atherosclerosis [13]. Unfortunately, the occurrence of ICH remains low in the American and European population [14,15,16,17]. Scientific publications regardingthe prevalence of ICH in Central-Eastern Europe are scarce, especially when it comes to those living in rural areas.

Poland was a developing country that underwent a political transformation, as did most countries in Central-Eastern Europe. Therefore, it is possible to assess the CVH for this part of Europe using Poland’s example. Research has shown that the risk of CVD is five times lower among people with ICH than that ofpeople with poor CVH [11,14,18]. Therefore, identification of people with potentially modifiable barriers in reaching ideal CVH should be a priority for public health. 

Therefore, in this study, we aimed to estimate the prevalence of ideal and poor CVH in the Polish adult population based on the example of inhabitants of the Janów district, in Lubelskie Voivodeship (Eastern Poland) following the criteria of the AHA. Our secondary objective was to assess the relationship between place of residence and gender and ICH.

Data of the Central Statistical Office, the Local Data Bank (GUS, BDL) for 2012 indicate that cardiovascular diseases were responsible for 58.1% of all deaths in Janów district, while in Poland in the analyzed period, mortality for the same reasons accounted for 46.1% of all deaths [19]. Correspondingly, the analysis of many indicators of the socioeconomic situation of people living in this area was also very unfavorable, similarly when compared to the general population, i.e., a higher rate of people with primary education in relation to the general population (27.55% vs. 23.2%, respectively) [20], and an unemployment rate of 15.6%, compared with an unemployment rate of 14% for the country [21]. The indicator regarding the number of people in families who receive social assistance for Janów district was relatively high—the social welfare system covered as much as 14.6% of the population (for comparison, the average in Lubelskie Voivodeship is 9.4%) [22].

## 2. Materials and Methods

### 2.1. Study Design and Participants

**Figure 1:**
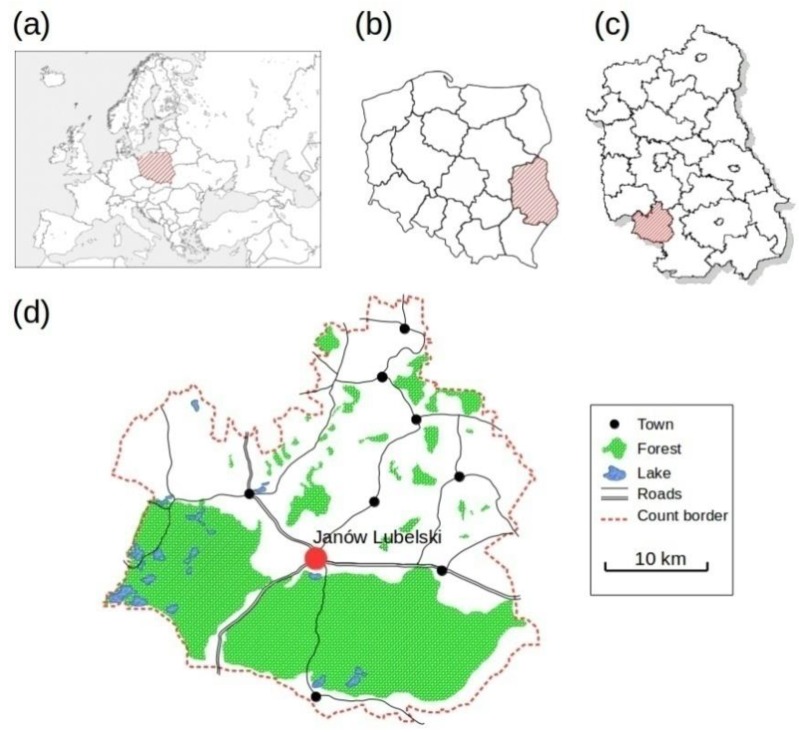
Approximate residential locations of participants, Janów district, Poland. (**a**) Europe; (**b**) Poland; (**c**) Lubelskie Voivodeship; (**d**) Janów district.

A prevention and health promotion program with respect to the awareness of CVDs, entitled “Follow Your Heart” (“Weź sobie zdrowie do serca”), was conducted between 06.14.2015 and 03.20.2016 among the residents of Janów district, Lubelskie Voivodship, Eastern Poland (Figure 1). The implementation of the above project, as a part of the Program PL 13 “Limiting social inequities in health” funded by the Norwegian Financial Mechanism 2009–2014 through a tender procedure, was possible in local communities with high standardized mortality rates, including cardiovascular reasons as causes. The list of districts eligible to participate in the competition was developed based on the highest standardized mortality ratios (SMRs) for the period 2009–2011 in terms of: cancer, cardiovascular diseases, respiratory diseases, digestive system diseases, external causes, and total mortality. Janów district was found on this list, taking the third position (SMR = 1.357) among the 38 districts characterized by the highest mortality rates standardized CVD [23].

47,500 people lived in Janów district in the period preceding the study [24]. The group participating in the project included inhabitants aged 35–64, accounting for18,827 people, according to population and electoral records of Janów district. The recruitment of research participants and promotion of the project was conducted through district and municipal local councils and cooperating institutions (religious associations, workplaces, associations and public-benefit organizations) and by direct telephone calls to prospective participants. To ensure equal access to the project for the prospective participants, 15 points were organized in Janów district, where respondents could register (14 mobile points, traveling in different towns; and one stationary point, located in the City Hospital, which also had a coordinating function). As many as 4040 people accepted the invitations to participate in the project; the general participation rate in our research was 21.45% of the eligible population. All participants agreed to undergo tests and provided their consent to participate in this study. Following were the exclusion criteria: previous cardiovascular incident (heart attack or cerebrovascular accident) cardiomyopathy, chronic kidney disease, pregnancy, being unable to provide consent, immobility, and living in nursing homes or in prisons. A total of 4040 people participated, among which139 participants were excluded from the study due to their prior history of cardiovascular incidents (heart attacks or cerebrovascular accidents). Data on the incidence of cardiovascular events were collected in a direct interview. In addition, each of the respondents had a resting ECG, evaluated and interpreted by a Doctor of Medicine. This study was approved by the Bioethical Commission of the Medical University in Lublin (no. KE-0254/112/2014) and was conducted in accordance with the Declaration of Helsinki. All the final participants provided written informed consent.

### 2.2. Data Collection

The team collecting the data consisted of trained nurses. All participants filled in the questionnaire and underwent anthropometric tests (weight and height), physiological tests (two measures of blood pressure), and had their venous blood samples collected in order to analyze glucose and total cholesterol levels in blood serum, through fasting. The questionnaire included items concerned with the past and currently treated diseases.

### 2.3. ICHMetrics

Indicators of ICH and their components were calculated according to AHA guidelines, using cut-off indicators for adults (Table S1) [4].

#### 2.3.1. Assessment of Biological Components of ICH

Research participants had their blood pressure (BP) measured twice. BP was measuredonthe left arm using an electronic sphygmomanometer. The first measurement was performed after at least a 5-min rest, and the second was performed 15 min after the first measurement. The average of the two was then calculated. If they differed by more than 5 mm Hg, an additional measurement was performed after another 15 min, and the average of the three was used for further data analysis [5]. Ideal blood pressure was defined as a blood pressure of <120/<80 mm Hg without the use of any hypertensive medications. Intermediate blood pressure was defined at SBP 120–139 or DBP 80–89 mm Hg, or if the respondent was taken drug lowering the blood pressure, whereas poor blood pressure was defined as a value ≥140/≥90 mm Hg [4].

Blood samples were taken from the ulna vein, in the morning, after fasting and an all-night rest. This was done using a tube with a coagulation activator and a separation substance (granulate), which was delivered to the laboratory within 4 h. Plasma was separated by centrifugation at 3000 rpm and for 10 min. Blood serum was used to analyze the glucose and total cholesterol levels. Fasting blood glucose (FBG) was measured using the hexokinase enzymatic method, and total cholesterol concentration (TC) was measured by an enzymatic method. Both parameters were analyzed by using Advia 1800 or Advia 1200 apparatus using Siemens reagents. Analyses were performed in the Central Laboratory of Janów Lubelski Hospital.

Ideal capillary FBGis defined as FBG < 100 mg/dL (5.56 mmol/L)achieved by not consuming hypoglycemic medications. Intermediate glucose level isdefined as FBG of 100–125 mg/dL (5.56–6.96 mmol/L) or >100mg/dL (5.56 mmol/L) achieved by not taking hypoglycemic medications. Poor glucose level is defined as FBG ≥ 126 mg/dL (7 mmol/L). Ideal total cholesterol (TC) is defined as TC < 200 mg/dL (5.17 mmol/L)achieved by not taking medications aimed at lowering cholesterol levels. Intermediate cholesterol is defined as TC in the range 200–239 mg/dL (5.17–6.18 mmol/L) or <200 mg/dL (5.17 mmol/L)value, achieved by taking medicines aimed at lowering cholesterol levels. Finally, poor cholesterol level is defined as TC ≥ 240 mg/dL (6.21 mmol/L) value [4].

#### 2.3.2. Assessment of Behavioral Components of ICH

All respondents underwent anthropometric measurements of height and weight. Height was measured within an accuracy of 0.1cm by using an altimeter, and weight was recorded without shoes and other clothing, using a platform scale with an accuracy of 0.1 kg. BMIs were calculated, defined as the body mass (kg) divided by the squared height in meters (kg/m^2^) [25]. Ideal BMI is defined between 18.5 and 24.9 kg/m^2^, intermediate between 25 and 29.9 kg/m^2^, and poor BMI which is ≥30 kg/m^2^.

In the smoking category, a rating of the ideal was recorded if the respondent never smoked or quit smoking more than 12 months ago. Intermediate smoking is defined as smoking in the past but quit within 1–12 months prior to their participation in this study. Poor category involved everyday smoking (>1 cigarette a day or if the last cigarette was smoked during the last month).

Nutrition was evaluated using the Perinumeric Periodic Table questionnaire according to Starzyńska [26], investigating the nutrition pattern in the last 7 days. The questionnaire consisted of six questions which were linked to the number of meals per day (4–5 vs. 3 vs. less); number of meals incorporating animal protein (all meals vs. 75% of meals vs. smaller number of meals); frequency of consumption of milk and of milk products (every day in 2 meals vs. every day in at least 1 meal, and in 50% of days in 2 meals vs. more rarely); frequency of consumption of raw fruit and vegetables (every day in at least 3 meals vs. every day in at least 2 meals vs. more rarely); frequency of consumption of cooked fruit and vegetables (everyday vs. in 75% of days vs. more rarely); and the frequency of consumption of wholemeal bread, wheat, and pulses (every day at least one of the mentioned products vs. in 75% of days one of the mentioned products vs. more rarely).

The answers given by the respondents were rated in points. For example, on the first question (number of meals in a day), the respondent received 5 points for giving the first answer, 3 points for the second, and no points (0) for the last. Further questions were marked as follow: 5 points for the first answer, 2 points for the second answer, and no (0) points for the last answer. According to the AHA recommendations for nutrition in ideal cardiovascular health, an appropriate number of points was attributed to each response given [4].The maximum number of points awarded was 30 points. Those who obtaineda score in between 30 and 21 points were considered to have a healthy diet, with intermediate being those participants with points ranging from 20 to 13, and a poor diet for those respondents with 12 points or less. Reliability of questions in the scale was measured by the alfa Cronbach indicator and had a value of 0.73.The questionnaire by Starzyńska in confrontation with the method of quantitative evaluation of nutrition (according to the amount of energy supplied by the appropriately balanced meals and the content of protein, fat, carbohydrates, vitamins and minerals designed by a computer program) exhibits high cohesion in terms of reproducibility of provided evaluation [27].The assessment of physical activity was based on a question asking if the respondent participated in regular physical activity for 30 min at least 5 times a week. Physical activity ≥150 min a week was defined as ideal, and physical activity <150 min a week was defined as poor. The assessment did not evaluate physical activity in the intermediate category.

#### 2.3.3. Cumulated Index of Global ICH

From particular behavioral components and biological components described above, we created a cumulated index of ICH in accordance with AHA guidelines. Depending on the number of components (0–2, 3–4, or 5–7), ICH metrics of the respondents were qualified as poor, intermediate, or ideal.

### 2.4. Statistical Analysis

Data are expressed as the mean± standard deviation or as median (interquartile range, IQR), as appropriate. The Shapiro Wilk test was used to assess conformity with a normal distribution. Mean age between the two groups was compared using Student’s *t*-test. Categorical variables were analyzed using the χ^2^ test or Fisher’s exact test, as appropriate. Logistic regression was used to investigate the relationships between components of ICH (ideal category), ideal behavioral and ideal biological component, and place of residence (rural or urban). The two models were fitted: (1) with place of residence, and (2) model one, additionally adjusted for age, education level, marital status, and gender (in the case when the analysis was performed for all participants).

Due to the significant differences (or interactions) in the distribution of ICH components and ICH between male and females, we performed analysis separately for men and women. The results of logistic regression were presented as OR with 95% confidence interval (95% CI). Next, we used a Poisson regression model to compare the mean values of the ICH component between rural and urban residents. All statistical analyses were performed using the SPSS software version 22.0 (IBM, New York City, NY, USA). *p-*Values less than 0.05 were considered significant.

## 3. Results

### 3.1. General Characteristics of Participants

The average age of participants was found to be 52.11 ± 8.15 years, with city residents being slightly older (52.65 ± 8.41 years) than those of rural areas (51.85 ± 8.01 years) *p* = 0.004. Males constituted 41.1% (*n* = 1603) of the researched population. Table 1 and Table S2 present the socio-demographic characteristics and ICH components of the respondents, by place of residence (urban or rural) and gender. City residents were better educated, took antihypertensive and lipid-lowering medication more often, and smoked more frequently than the residents of rural areas. However, the percentages of participants from rural areas with ideal body mass, ideal diet, ideal blood pressure, and ideal glucose levels were found to be significantly lower compared to participants leaving in urban areas.

ICH was found in 5.4% of the participants, with the advantage going to urban residents compared to the participants from rural areas (6.3% vs. 5.0%) *p* = 0.02. The breakdown of CVH according to gender shows that CVH is present significantly more frequently in women than in men (7.6% vs. 2.2%) *p* < 0.001.

### 3.2. Participants’ Characteristics by CVHStatus

Table 2 presents the comparison between socio-demographic traits chosen for the diet questionnaire and the use of the therapy in global ICH categories in the group. ICH, in the residents of both rural and urban areas, was linked to a younger age, higher education, was more prevalent among women, and was also linked to diet (number of recommended meals a day, occurrence of milk, cheese, wholemeal bread, wheat, and pulses), and with the treatment of hypertension, diabetes, and hypercholesterolemia. Indicators such as living alone or consumption of protein products differentiated the categories of ideal health only in the case of rural areas residents.

### 3.3. The Relation between the Place of Residence and Each Metric of CVH

Table 3 presents the relation between the place of residence and ICH components in the whole sample and by gender. After adjusting for age, marital status, education, and gender, five of seven items (smoking, BMI, healthy diet, arterial blood pressure, and FBG) were tightly linked to the place of residence. Residents of rural areas had a 22% lower likelihood of an ideal BMI than that of residents of urban areas (OR = 0.78; 95% CI: 0.66–0.92); the likelihood of rural residents of having an ideal diet was reduced by 29% when compared with urban residents (OR = 0.71; 95% CI: 0.54–0.94); the likelihood of ideal arterial blood pressure of rural residents of was reduced by 29% when compared with urban residents (OR = 0.71; 95% CI: 0.56–0.89); and the likelihood of rural residents of having ideal glucose levels were reduced by 28% when compared with urban residents (OR = 0.72; CI: 0.63–0.84).

In the case of smoking, we obtained contradictory results with the occurrence of non-smokers being 35% more likely in rural areas in comparison to the residents of urban areas (OR = 1.35; 95% CI: 1.17–1.57). For the analyses based on gender, smoking status, BMI, healthy diet, arterial blood pressure, and FBG, the data related to women showed statistical significance and correlation. However, in men, only the ideal glucose levels were significantly linked to the place of residence.

### 3.4. The Relation between the Place of Residence and Global, Behavioral, and Biological Components of CVH

Table 4 presents the association between the place of residence and the ideal global ICH (5–7 items) and behavioral and biological components. Residents of rural areas had a 25% lower instance of global ICH than the residents of urban areas(OR = 0.72; 95% CI: 0.53–0.98). A greater reduction in the likelihood for global ICH was found in women (OR = 0.62; 95% CI: 0.44–0.87), whereas in men, this association was not found to be significant. None of the behavioral and biological components were significantly related to the place of residence, in both men and women.

## 4. Discussion

In this study, we aimed to estimate the prevalence of the seven CVH metrics—four behavioral and three biological—and we also estimated the accumulated CVH indicators according to the criteria recommended by the AHA using a cross-sectional analysis of data obtained from 3901 people aged between 35 and 64 years from the Janów district in Eastern Poland. We also studied whether the place of residence (urban or rural areas) and gender determines the level achieved with respect to ICH. Our study showed that the place of residence is significantly related to the ideal global ICH. Respondents living in rural areas had a lower chance of having the ICH according to the AHA criteria.

In Poland, two major studies on representative samples have been carried out, evaluating the prevalence of risk factors for cardiovascular diseases. These were WOBASZ, a multicenter nationwide study of the Polish population’s health and Polish part of the project HAPIEE (Health, Alcohol and Psychosocial Factors In Eastern Europe). The WOBASZ study was conducted among a representative sample of the general population, whereas the HAPIEE study included a representative sample for the city of Cracow [28,29]. In our study, we had a lower percentage of smokers and a lower percentage of participants with hypercholesterolemia among men and women than in the HAPPIE and WOBASZ studies. Also, women we examined had lower blood pressure values than in the HAPIEE and WOBASZ studies, while men were characterized by lower blood pressure values than men tested in the HAPPIE project, but higher than in the national sample. Among the surveyed women and men living in Janów district, the percentage of obese people was higher than in the studied population of Cracow and general one [8]. It should be noted, however, that our study was conducted about 10 years later and in broader age groups. Therefore, it is not surprising that the frequency of ICH in terms of 5–7 factors in the researched Polish population was found to be very low, and pertained to only 5.4% of the people. Of those researched, only 0.1% achieved 7 components of ICH metrics, which is considered to be minor. Similar results were found by Manczuk et al. [30]. However, in their research, performed on 10,687 people in Kielce, South-Eastern Poland, none of the respondents reached all seven ideal ICH components.

The frequency of occurrence of all seven CVH indicators is low all over the world, and varies from 0.2% to 15%, depending on geographical location, age, gender, ethnic background, and education level [16,31,32,33,34]. The rate of achieving ICH results (5–7 ideal metrics) in the researched Polish population was higher than in the Iranian population [35], in the adult population of Republic of Serbia, as well as the adult population of Bosnia and Herzegovina [10]. However, their results were lower than the results of those from Brazil [36], the Canadian research conducted by Maclagan et al. [37], the research of the population of Peru [34], and samples of the American population [38,39].

Understanding the potential of cardiovascular health in the adult population in terms of society was implemented by investigating 7 significant factors: four behavioral components and three biological components, enabling improved planning concerning the health policy by creating local conditions and developing health programs tailored to the needs of the community to which it is addressed.

This study suggests that the percentage of people with ICH living in rural areas was significantly lower than in those living in the urban areas (5.0% vs. 6.3%). Moreover, living in rural areas was linked to a lower chance of reaching an ideal BMI, having an ideal diet, and ideal arterial blood pressure. However, it was more likely for those residing in rural areas to be non-smokers than residents of urban areas.

Observed imbalances in CVH are undoubtedly correlated with a higher number of males living in rural areas, as well as a lower rate of people with higher education living in rural areas, which might cause the guidelines concerning a healthy lifestyle to be ignored. Living in rural areas may also be connected to a limited access to health services. In a study conducted in Peru, the average number of ICH components in the rural area of Tumbes was found to be 2.82, which was significantly lower compared to the residents of the city of Lima [34].Socioeconomic inequalities in overall mortality in a prospective population study (in the group of 16,812 men and 19,180 women aged 45–69) in the Czech Republic, Russia, Poland and Lithuania evidently support the results of the HAPIEE study. The results emphasize the importance of all the tested socioeconomic position (SEP) elements, significant for understanding mortality inequality in the countries of Central and Eastern Europe [40]. In accordance with the existing research [17,37,41,42,43], we observed that women were more likely to have ICH compared to men. Studies including this suggest that there should be additional effort put into the promotion of primary prevention of CVD [15].

Among the behavioral components of ICH metrics, the best factor assisting the achievement of ICH was not smoking (63.1% of the researched), and the most difficult with regard to maintaining CVH was practicing an ideal healthy diet, which was only achieved by 6.5% of respondents. These results have been confirmed by the systematic review conducted by Younus et al. [32], where the prevalence of the ideal classification of non-smokers in the analyzed population was higher, and the poorest indicator was the diet. The above observation of such a low maintenance index of Ideal Healthy Diet in conducted research, as well as other authors, is a particularly important issue considering the HAPIEE study. The findings from the HAPIEE study confirm the hypothesis that an unhealthy diet play an important role in high mortality due to CVD in countries in Central and Eastern Europe (CEE) and the Former Soviet Union (FSU) [44]. Of all the biological components of ICH metrics, only 10.3% in the researched group had ideal blood pressure, out of which a greater percentage were living in the urban areas rather than in rural areas.

The idea of strengthening CVH should be applied to the health policy of the country, especially via the prism of primary prevention aimed at the weakest links of the society, namely people with a lower education, those living in rural areas, and males. Our discovery could have a potential impact on individual and public health. Intensified efforts aimed at taking behavioral factors (especially diet and body mass) into consideration are necessary, as well as the detection and control of biological factors, especially measuring of arterial blood pressure. The relation between ICH and residing in urban areas versus rural areas has been shown. Efforts aimed at the promotion of CVH and prevention of CVDs should especially focus on residents of rural areas.

## 5. Strengths

A key aspect of our project is an investigation concerning the largest number of respondents with the highest adverse mortality (SMR) rates in 2009–2011 due to cardiovascular diseases, including rural populations. The sizable sample allows us to better understand the specificity of the studied phenomena, especially in their multifactorial conditions. Another important aspect of our project includes its implementation in a local community characterized by lower socioeconomic status compared to the population of the region and the country in terms of: low education, high unemployment and a higher poverty rate.

## 6. Study Limitations

Our research has certain limitations which should be considered. First, this study was concerned with the population of South-Eastern Poland, in addition, the selection of participants was devoid of randomization and stratification concerning the sample with respect to their age and gender; also, the data on cardiovascular events and heart disease of the participants (which were the exclusion criteria) were collected on the basis of direct interview, without examining the medical history of the patient. Future studies should be conducted on a representative sample for Polish population. In addition, the cross-sectional design of the presented study and the analysis of results reduces its strength for cause-and-effect reasoning, showing only a tendency of the relationship between biological and behavioral factors and CVD.

Another limitation is the questionnaire assessing physical activity and diet. In terms of physical activity, the participants were asked only one question, and because of this, there was no middle category in ICH. The diet questionnaire did not include a question concerning fish and seafood consumption, which generally results from the low popularity of fish and seafood among the Polish population, as well as the high price of these products, which are rarely eaten by the less affluent rural areas residents. In Poland, people consume a lot of potatoes, pork, butter, cereal preparations, vegetables, sugar, and small amounts of fruit, veal, beef, milk, and fish [45]. Therefore, our diet questionnaire was customized to the dietary trends in Poland. However, neither ESC, nor AHA explicitly recommend a questionnaire for the assessment of diet; therefore, we used a questionnaire which contains a large part of questions in accordance with the ESC and AHA dietary guidelines, which indicate the dietary patterns desirable from the perspective of prevention of cardiovascular risk.

Our analysis concerning the prevalence of ICH consisted of the qualification of 5–7 factors as a measurement of ICH. Many available studies qualify 6–7 factors as the ICH measurement according to the AHA criteria [15]. Observed differences in the qualification approach demand standardization for a better understanding of the real determinants of ICH. It also needs to be observed that the creation of ICH results is-based upon the usage of binary variables, with the assumption that all health behaviors and factors affect the final result.

## 7. Conclusions

Our research proved that important trends in health behaviors and biological factors were linked to the maintenance of CVH in Poland within the adult population, as exemplified by the population of Janów disctict, in Lubelskie Voivodeship. Although the levels of physical activity, nonsmoking, or healthy diet can be improved, the problems of obesity, hypertension, and diabetes worsen, which demands greater attention. Based on the visible positive changes of certain health behaviors, what is needed is targeted political and program intervention to increase all factors. This includes physical activity and diet quality, which shall prospectively improve the state of CVH in the Polish people, and the frequency of occurrence of CVD shall decrease.

We expect that the CVH index will be a useful tool for the whole of society, clinicians, researchers, as well as the policymakers interested in the monitoring of CVH, in order to decrease the level of the social burden caused by CVDs in Poland.

## Figures and Tables

**Table 1:** Characteristics of the researched group according to their place of residence.

Characteristics of Population	Rural Areas		Urban Areas		Total		*p*-Value
	*n*	%	*n*	%	*n*	%	
	2605	66.78	1296	33.22	3901	100	<0.001
Male	1109	42.6	494	38.1	1603	41.1	0.08
Age	51.85±8.01		52.65±8.41		52.11±8.15		0.004
University education	314	12.1	442	34.1	756	19.4	<0.001
Single respondents (bachelorette/bachelor, widow/widower, divorcee)	286	11	192	14.7	478	12.3	0.001
Blood pressure medication	665	25.5	393	30.3	1058	27.1	0.002
Glucose-lowering medication	102	3.9	67	5.2	169	4.3	0.07
Cholesterol-lowering medication	240	9.2	150	11.6	390	10	0.02
Smoking							
Poor	396	15.2	224	17.3	620	15.9	0.02 ^#^
Intermediate	526	20.2	294	22.7	820	21	0.005 ^##^
Ideal	1683	64.6	778	60	2461	63.1	
Body Mass Index							
Poor	1000	38.4	403	31.1	1403	36	<0.001 ^#^
Intermediate	1024	39.3	538	41.5	1562	40	<0.001 ^##^
Ideal	581	22.3	355	27.4	936	24	
Physical Activity							
Poor	1470	56.4	764	59	2234	57.3	
Intermediate	-	-	-	-	-	-	0.13 ^##^
Ideal	1135	43.6	532	41	1667	42.7	
Healthy Diet							
Poor	2293	88	1091	84.2	3384	86.7	0.002 ^#^
Intermediate	165	6.3	98	7.6	263	6.7	0.002 ^##^
Ideal	147	5.6	107	8.3	254	6.5	
Fasting Total Cholesterol							
Poor	847	32.5	307	23.7	1154	29.6	<0.001 ^#^
Intermediate	852	32.7	501	38.7	1353	34.7	0.078 ^##^
Ideal	906	34.8	488	37.7	1394	35.7	
Blood Pressure							
Poor	1429	54.9	597	46.1	2026	51.9	<0.001 ^#^
Intermediate	935	35.9	538	41.5	1473	37.8	<0.002 ^##^
Ideal	241	9.3	161	12.4	402	10.3	
Fasting Serum Glucose							
Poor	256	9.8	90	6.9	346	8.9	<0.001 ^#^
Intermediate	806	30.9	349	26.9	1155	29.6	<0.001 ^##^
Ideal	1543	59.2	857	66.1	2400	61.5	
Cardiovascular health metrics							
Poor cardiovascular health (0–2 ideal metrics)	1462	56.1	672	51.9	2134	54.7	0.02 ^#^
Intermediate cardiovascular health (3–4 ideal metrics)	1014	38.9	542	41.8	1556	39.9	0.07 ^##^
Ideal cardiovascular health (5–7 ideal metrics)	129	5	82	6.3	211	5.4	
No. of ideal cardiovascular health metrics							
0	116	4.5	45	3.5	161	4.1	0.1
1	509	19.5	223	17.2	732	18.8	
2	837	32.1	404	31.2	1241	31.8	
3	680	26.1	348	26.9	1028	26.4	
4	334	12.8	194	15	528	13.5	
5	101	3.9	66	5.1	167	4.3	
6	24	0.9	15	1.2	39	1	
7	4	0.2	1	0.1	5	0.1	

^#^ Ideal vs. poor vs. intermediate; ^##^ Ideal vs. poor + intermediate.

**Table 2:** Characteristics of the group according to their cardiovascular health.

Variable	Rural Areas						*p*-Value	Urban Areas						*p*-Value
	Poor		Intermediate		Ideal			Poor		Intermediate		Ideal		
	*n*	%	*n*	%	*n*	%		*n*	%	*n*	%	*n*	%	
	1462	56	1014	39	129	5		672	52	542	42	82	6	
Age	53.17 ± 7.69		50.66 ± 8.08		46.25 ± 7.28		<0.001	54.06 ± 8.0		51.68 ± 8.54		47.44 ± 7.94		<0.001
Male	697	47.67	385	37.97	27	20.93	<0.001	289	43.01	196	36.16	9	10.98	<0.001
University education	145	9.92	139	13.71	30	23.26	<0.001	190	28.27	217	40.04	35	42.68	<0.001
Single respondents	171	11.7	111	10.95	5	3.88	0.02	101	15.03	84	15.5	6	7.32	0.14
Number of meals in a day _Ideal_	730	49.93	555	54.73	84	65.12	<0.001	338	50.3	305	56.27	54	65.85	0.009
Protein consumption _Ideal_	150	10.26	137	13.51	23	17.83	0.005	42	6.25	48	8.86	7	8.54	0.21
Milk and cheese consumption _Ideal_	115	7.87	120	11.83	25	19.38	<0.001	52	7.74	52	9.59	20	24.38	<0.001
Fruit and vegetables _Ideal_	57	3.90	72	7.10	23	17.83	<0.001	45	6.7	74	13.65	24	29.27	<0.001
Wholemeal bread, groans and pulses _Ideal_	218	14.91	218	21.5	39	30.23	<0.001	142	21.13	164	30.26	41	50	<0.001
Treatment for blood pressure	472	32.28	187	18.44	6	4.65	<0.001	258	38.39	128	23.62	7	8.54	<0.001
Treatment for diabetes	90	6.16	12	1.18	0	0	<0.001	56	8.33	11	2.03	0	0	<0.001
Treatment of cholesterol	198	13.54	42	4.14	0	0	<0.001	106	15.77	43	7.93	1	1.22	<0.001

**Table 3:** The relation between the place of residence and ICH components altogether, and according to gender.

	Smoking (Ideal)		Body Mass Index (Ideal)		Physical Activity (Ideal)		Healthy Diet (Ideal)		Fasting Total Cholesterol (Ideal)		Blood Pressure (Ideal)		Fasting Serum Glucose (Ideal)	
	OR	95% CI	OR	95% CI	OR	95% CI	OR	95% CI	OR	95% CI	OR	95% CI	OR	95% CI
All														
Urban areas	1		1		1		1		1		1		1	
Rural areas	1.21 ^A^	[1.06–1.39] *	0.71 ^A^	[0.60–0.83] ***	1.12 ^A^	[0.98–1.28]	0.66 ^A^	[0.51–0.86] **	0.85 ^A^	[0.75–1.001]	0.67 ^A^	[0.54–0.84] **	0.73 ^A^	[0.63–0.84] ***
	1.35 ^B^	[1.17–1.57] ***	0.78 ^B^	[0.66–0.92] **	1.05 ^B^	[0.91–1.21]	0.71 ^B^	[0.54–0.94] **	0.88 ^B^	[0.76–1.02]	0.71 ^B^	[0.56–0.89] **	0.72 ^B^	[0.63–0.84] ***
Male														
Urban areas	1		1		1		1		1		1		1	
Rural areas	1.07 ^A^	[0.87–1.32]	1.03 ^A^	[0.78–1.36]	1.13 ^A^	[0.92–1.40]	0.66 ^A^	[0.40–1.09]	0.81 ^A^	[0.65–1.004]	0.73 ^A^	[0.44–1.21]	0.69 ^A^	[0.55–0.86] ***
	1.14 ^B^	[0.92–1.42]	0.98 ^B^	[0.72–1.30]	1.15 ^B^	[0.92–1.43]	0.75 ^B^	[0.45–1.27]	0.81 ^B^	[0.65–1.02]	0.72 ^B^	[0.43–1.21]	0.67 ^B^	[0.54–0.85] ***
Female														
Urban areas	1		1		1		1		1		1		1	
Rural areas	1.49 ^A^	[1.23–1.80] ***	0.57 ^A^	[0.46–0.69] ***	1.09 ^A^	[0.91–1.30]	0.68 ^A^	[0.50–0.92] *	0.84 ^A^	[0.70–1.02]	0.65 ^A^	[0.50–0.83] *	0.76 ^A^	[0.64–0.91] **
	1.56 ^B^	[1.27–1.90] ***	0.65 ^B^	[0.52–0.80] ***	0.98 ^B^	[0.81–1.18]	0.70 ^B^	[0.50–0.97] *	0.90 ^B^	[0.74–1.10]	0.70 ^B^	[0.53–0.90] **	0.77 ^B^	[0.63–0.93] **

^A^ Unadjusted; ^B^ Adjusted for age, sex, marital status, and education; Statistical significance is indicated by * *p* ≤ 0.05, ** *p* ≤ 0.01 and *** *p* ≤ 0.001.

**Table 4:** The relation between the place of residence and the ideal global ICH, behavioral, and biological components, and according to gender.

	All 5–7 Items (Ideal)		Behavioral Component (Ideal)		Biological Component (Ideal)	
	OR	95% CI	OR	95% CI	OR	95% CI
All						
Urban areas	1		1		1	
Rural areas	0.73 ^A^	[0.55–0.98] *	0.97 ^A^	[0.75–1.20]	0.95 ^A^	[0.83–1.08]
	0.72 ^B^	[0.53–0.98] *	1.04 ^B^	[0.81–1.32]	0.95 ^B^	[0.82–1.09]
Male						
Urban areas	1		1		1	
Rural areas	1.37 ^A^	[0.64–2.94]	1.53 ^A^	[0.91–2.59]	0.97 ^A^	[0.79–1.21]
	1.33 ^B^	[0.61–2.92]	1.67 ^B^	[0.97–2.87]	0.95 ^B^	[0.77–1.19]
Female						
Urban areas	1		1		1	
Rural areas	0.64 ^A^	[0.47–0.89] **	0.86 ^A^	[0.66–1.11]	0.94 ^A^	[0.79–1.11]
	0.62 ^B^	[0.44–0.87] **	0.90 ^B^	[0.68–1.18]	0.95 ^B^	[0.79–1.14]

^A^ Unadjusted; ^B^ Adjusted for age, sex, marital status, and education; Statistical significance is indicated by * *p* ≤ 0.05, ** *p* ≤ 0.01; Behavioral component: smoking, physical activity, BMI, and diet; Biological component: blood pressure, blood glucose, and cholesterol levels.

## Supplementary Materials

The following are available online at http://www.mdpi.com/1660-4601/15/11/2388/s1, Table S1: The definition of cardiovascular health in our research (according to AHA criteria), Table S2: Characteristics of the researched group according to their gender.
