# Supplementary material for: The Status of Cardiovascular Health in Rural and Urban Areas of Janów Lubelski District in Eastern Poland: A Population-Based Study
Source: Int J Environ Res Public Health. 2018 Oct 28;15(11):2388. doi: 10.3390/ijerph15112388 (PMC6266283; doi:10.3390/ijerph15112388)
Supplement: Supplementary file 1 [file ijerph-15-02388-s001.pdf]

International Journal of
Environmental Research and Public Health

MDPI

Supplementary File

The Status of Cardiovascular Health in Rural and Urban Areas of Janów Lubelski District in Eastern Poland: a Population-Based Study

Grzegorz Józef Nowicki*, Barbara Ślusarska, Honorata Piasecka, Agnieszka Bartoszek, Katarzyna Kocka and Alina Deluga

Table S1. The definition of cardiovascular health in our research (according to AHA criteria).

Metric	Definition
**Behavioural component**	
Smoking	
Poor	Currently smoke (at least 1 cigarette a day or the last cigarette was burned in the last month)
Intermediate	Smoked in the past and quit smoking 1—12 months ago
Ideal	Never smoked or quit smoking> 12 months ago
Body mass index	
Poor	≥ 30 kg/m^2
Intermediate	25—29.9 kg/m^2
Ideal	18.5—24.9 kg/m^2
Physical activity	
Poor	< 150 minutes of physical activity / week
Intermediate[#]	-
Ideal	≥ 150 minutes of physical activity / week
Healthy diet score	
Poor	≥ 12 points in the diet test
Intermediate	20—13 points in the diet test
Ideal	30—21 points in the diet test
**Biological component**	
Total cholesterol	
Poor	≥ 240 mg/dl (6.21 mmol/L)
Intermediate	200—239 mg/dl (5.17–6.18 mmol/L) or < 200 mg/dl (5.17 mmol/L) and intake of cholesterol-lowering medications
Ideal	< 200 mg/dl (5.17 mmol/L) and not taking cholesterol-lowering medications
Blood pressure	
Poor	SDP ≥ 140 mm Hg and DBP ≥ 90 mm Hg
Intermediate	SBP 121—139 and DBP 80—89 or intake of hypertensive
Ideal	SBP ≤ 120 mm Hg and DBP ≤ 80 mm Hg without hypotensive treatment

Table S1. *Cont.*

Metric	Definition
**Biological component**	
Fasting plasma glucose	
Poor	≥ 126 mg/dl (≥ 7 mmol/L)
Intermediate	100—125 mg/dl (5.56 – 6.96 mmol/L) or > 100 mg/dl (>5.56 mmol/L) and intake of hypoglycaemic medications
Ideal	< 100 mg/dl (< 5.56 mmol/L) and not taking hypoglycaemic medications

[#]Value not taken into consideration

Table S2. Characteristics of the researched group according to their gender.

**Characteristics of population**	**Female**		**Male**		*P*-value
	n	%	n	%	
	2298	58.9	1603	41.1	0.08
Smoking					
Poor	262	11.4	358	22.3	< 0.001#
Intermediate	369	16.1	451	28.1	< 0.001##
Ideal	1667	72.5	794	49.5	
Body Mass Index					
Poor	814	35.4	589	36.7	< 0.001#
Intermediate	828	36	734	45.8	< 0.001##
Ideal	656	28.5	280	17.5	
Physical Activity					
Poor	1383	60.2	851	53.1	< 0.001#
Intermediate	-	-	-	-	
Ideal	915	39.8	752	46.9	
Healthy Diet					
Poor	1946	84.7	1438	89.7	< 0.001#
Intermediate	166	7.2	97	6.1	< 0.001##
Ideal	186	8.1	68	4.2	
Fasting Total Cholesterol					
Poor	701	30.5	453	28.3	0.2#
Intermediate	799	34.8	554	34.6	0.12##
Ideal	798	34.7	596	37.2	
Blood Pressure					
Poor	1010	44	1016	63.4	< 0.001#
Intermediate	955	41.6	518	32.3	< 0.001##
Ideal	333	14.5	69	4.3	
Fasting Serum Glucose					
Poor	192	8.4	154	9.6	0.06#
Intermediate	658	28.6	497	31	0.02##
Ideal	1448	63	952	59.4	
Cardiovascular health metrics					
Poor cardiovascular health (0–2 ideal metrics)	1148	50	986	61.5	<0.001#
Intermediate cardiovascular health (3–4 ideal metrics)	975	42.4	581	36.2	< 0.001##
Ideal cardiovascular health (5–7 ideal metrics)	175	7.6	36	2.2	

Table S2. *Cont.*

**Characteristics of population**	**Female**		**Male**		*P*-value
	n	%	n	%	
	2298	58.9	1603	41.1	0.08
No. of ideal cardiovascular health metrics					
0	59	2.6	102	6.4	<0.001
1	383	16.7	349	21.8	
2	706	30.7	535	33.4	
3	611	26.6	417	26	
4	364	15.8	164	10.2	
5	136	5.9	31	1.9	
6	34	1.5	5	0.3	
7	5	0.2	0	0	

[#]Ideal *vs.* poor *vs.* intermediate; [##]Ideal *vs.* poor + intermediate
